# Beyond Confidence: A Narrative Review Assessing Educational Outcomes in Simulation-Based Obstetric Training for Medical Students

**DOI:** 10.7759/cureus.117592

**Published:** 2026-10-07

**Authors:** Ioana G Visan, Aida Petca

**Affiliations:** 1 Obstetrics and Gynecology, Carol Davila University of Medicine and Pharmacy, Bucharest, ROU; 2 Obstetrics and Gynaecology, CF2 Clinical Hospital, Bucharest, ROU

**Keywords:** clinical medical student, high fidelity simulation training, obstetrics&gynaecology, simulation in medical education, vaginal birth

## Abstract

Simulation-based education has become an increasingly important component of undergraduate medical training, particularly in obstetrics, where exposure to rare but high-risk emergencies may be limited during clinical rotations. Simulation provides a safe environment in which students can acquire procedural skills, improve decision-making, and develop confidence before encountering critical clinical situations. However, educational effectiveness is assessed differently across simulation-based obstetric training studies.

This narrative review examines the educational outcomes and assessment approaches used in simulation-based obstetric training among medical students. Particular attention is given to knowledge acquisition, technical and procedural performance, non-technical skills, self-confidence and self-efficacy, learner satisfaction, retention of acquired competencies, and transfer to clinical practice. The review also explores the assessment tools used to measure these outcomes, including structured checklists, global rating scales, objective structured clinical examination-based approaches, and investigator-developed instruments.

Current evidence suggests that simulation frequently improves learners' knowledge, perceived preparedness, and confidence. Nevertheless, subjective outcomes are often reported alongside, or sometimes in place of, objective measures of clinical competence. Furthermore, heterogeneity in assessment instruments and limited evaluation of long-term retention and clinical transfer make cross-study comparisons difficult.

By critically examining how educational outcomes are defined and measured, this review seeks to identify current limitations in simulation assessment and highlight priorities for future research, including greater use of validated objective measures, standardized outcome frameworks, delayed assessment, and evaluation of transfer from simulated to clinical environments.

## Introduction and background

Opportunities for medical students to participate directly in high-risk or time-sensitive clinical situations are often limited. Simulation helps address this constraint by providing a controlled setting in which clinical reasoning, procedural skills, communication, and decision-making can be practiced without relying exclusively on unpredictable clinical exposure. Current simulation-based approaches include physical task trainers and high-fidelity manikins, immersive or virtual modalities, and competency-oriented assessment frameworks [1-3].

Simulation has become an important educational strategy across medical training and, specifically, in obstetric education [4-6]. Undergraduate medical students must acquire essential skills related to childbirth and the recognition and initial management of obstetric emergencies. At the same time, their exposure to these situations during clinical rotations is inherently unpredictable. Emergencies such as postpartum hemorrhage, shoulder dystocia, eclampsia, cord prolapse, or acute fetal compromise require rapid recognition, structured decision-making, effective communication, and coordinated intervention. Their relatively low frequency and potential consequences for maternal and neonatal safety limit how much inexperienced learners can assume an active role in real clinical settings. Simulation therefore offers an opportunity to repeatedly rehearse both technical and non-technical aspects of obstetric care before these competencies are required in clinical practice [4-6].

Evidence supporting the educational value of obstetric simulation has grown steadily. Studies involving undergraduate medical students have reported improvements in knowledge, procedural performance, perceived preparedness, and self-confidence following simulation-based training [5,7-9]. A 2026 systematic review and meta-analysis similarly found greater post-training knowledge acquisition with simulation than with conventional teaching, while also highlighting marked heterogeneity in study design and outcome assessment and the limited evidence on long-term retention and clinical outcomes [9].

These findings are encouraging, but their interpretation depends heavily on how educational benefit is defined. Simulation studies use a broad range of outcome measures and assessment instruments, including satisfaction, perceived preparedness, knowledge, technical performance, teamwork, and communication [10,11]. Because these measures reflect different dimensions of learning, improvements in one domain should not automatically be taken as evidence of improvement in another.

Self-confidence deserves particular consideration in this context. Increased confidence is a relevant educational outcome because it may influence learner engagement, participation in clinical activities, and willingness to perform unfamiliar tasks [12]. Obstetric simulation studies have repeatedly demonstrated significant increases in student confidence following training [5,7,8]. Nevertheless, confidence should not be interpreted as synonymous with competence. Gottlieb et al. emphasized that confidence is optimally meaningful when appropriately calibrated with objectively demonstrated performance and proposed that both overconfidence and underconfidence may have important implications for clinical education and practice [12]. Consequently, assessment strategies relying predominantly on self-reported confidence or perceived competence may provide an incomplete representation of learning.

This distinction is particularly relevant in undergraduate obstetric education. Evidence on the transfer of learning from simulation to clinical practice remains limited [13], although recent obstetric simulation studies have begun to combine learner-reported outcomes with objective measures of performance [14]. Yet most studies still rely on immediate post-training assessment, and comparatively little is known about the durability of these gains or their transfer to real clinical practice [9,13].

This review therefore focuses on how learning is evaluated in simulation-based obstetric training for medical students. It considers knowledge, technical and non-technical performance, self-confidence and self-efficacy, confidence-competence alignment, retention, and transfer to clinical practice, with particular attention to the strengths and limitations of current assessment practices.

Literature search approach

Relevant literature was identified through searches of PubMed/MEDLINE and Scopus, complemented by screening the reference lists of pertinent reviews and primary studies. Search terms combined concepts related to simulation-based education, medical students, obstetric simulation and obstetric emergencies, clinical competence, confidence, self-efficacy, assessment, and skill retention. Priority was given to peer-reviewed English-language publications, with emphasis on recent evidence and studies directly relevant to undergraduate medical education. Because this was a narrative review, the search aimed to identify representative and conceptually informative evidence rather than provide an exhaustive systematic synthesis.

## Review

Educational outcomes in obstetric simulation: what are we measuring?

Simulation-based obstetric training can influence several aspects of learning simultaneously, from factual knowledge and procedural performance to teamwork, clinical reasoning, and self-confidence [9-11]. This breadth is one of its educational strengths, but it also makes it difficult to select and compare outcomes across studies.

Knowledge acquisition

Knowledge represents one of the most frequently assessed outcomes in simulation-based obstetric education. Pre- and post-intervention questionnaires, multiple-choice tests, and locally developed knowledge assessments are commonly used because they are relatively easy to administer and allow straightforward quantification of immediate educational gains [9,15]. Evidence suggests that simulation-based interventions can improve theoretical knowledge and applied clinical competencies [15]. In broader undergraduate medical education, structured experiential programs have similarly reported concurrent development of knowledge, skills, and confidence [16].

A recent systematic review and meta-analysis of simulation-based teaching for obstetric emergencies among healthcare students showed significantly greater knowledge acquisition after simulation than with conventional educational approaches. In the pooled analysis of 7 studies including 333 participants, simulation was associated with a standardized mean difference of 0.84 (95% CI 0.36-1.32; p = 0.0005), although substantial heterogeneity was present (I² = 75.1%). The authors also highlighted the limited evidence regarding long-term retention, emphasizing that improvements observed immediately after simulation cannot necessarily be assumed to persist over time [9].

Similar findings have been reported in undergraduate medical education. Young et al. implemented a transvaginal ultrasound simulation focused on evaluating first-trimester bleeding among third-year medical students. Mean knowledge scores increased from 58% before the intervention to 69% after simulation, while students also reported substantial improvements in their perceived comfort with ultrasound-based diagnosis and management [17]. This study illustrates a recurrent pattern within simulation research: objective knowledge outcomes are frequently assessed alongside subjective measures of confidence or comfort, although the relationship between these dimensions is not always explored [12,17].

Knowledge testing captures an important part of learning, but it does not show whether a student can recognize deterioration, prioritize actions, perform a procedure, or communicate effectively under pressure. Knowledge should therefore be interpreted as one component of competence rather than as a proxy for overall clinical performance [12,15].

Technical and procedural performance

A major advantage of simulation-based education is its capacity to evaluate what learners can actually perform rather than solely what they know. This distinction is particularly relevant in obstetrics, where many essential competencies involve procedural or psychomotor skills, including vaginal delivery, management of postpartum hemorrhage and shoulder dystocia, obstetric ultrasound, and repair of perineal trauma [14,17,18].

Objective procedural assessment is generally performed using task-specific checklists, structured observation tools, procedure completion measures, or global rating scales [10,11,14]. These approaches can capture whether predefined critical actions are completed and, in some cases, assess the quality, sequence, or efficiency of performance. However, instruments vary considerably across studies and are often developed locally for a specific simulation scenario. This variability limits direct comparison between educational interventions and raises questions regarding the validity and reproducibility of some reported performance outcomes [10,11].

Recent technological developments have expanded the possibilities for procedural assessment. Gosavi et al. compared virtual reality with mannequin-based simulation for teaching normal vaginal delivery to medical students in a cluster-randomized crossover study [18]. Both approaches were used to teach the same clinical procedure, while the virtual environment enabled domain-specific feedback on learner performance. Such platforms offer the possibility of capturing detailed information regarding procedural sequencing and task execution, potentially providing a more standardized form of assessment than conventional observer-based evaluation.

Nevertheless, technology-generated metrics should not automatically be equated with clinical competence. Their educational value ultimately depends on whether the measured actions correspond to clinically meaningful performance and whether competence demonstrated in the simulated setting can subsequently be transferred to patient care [11-13,18].

High-fidelity obstetric simulation has also been associated with increased self-efficacy in maternity nurses [19], illustrating that subjective gains may occur without direct measurement of technical performance. Pairing self-assessment with observed performance can therefore provide a more informative picture than either measure alone [20].

Beyond procedural execution, obstetric simulation can also reveal how learners recognize deterioration, prioritize interventions, and escalate care [14,20]. These higher-order decisions are often embedded within task-specific checklists rather than assessed as a separate construct. A checklist can confirm that an action occurred, but it may not establish whether the learner understood why it was required or could adapt the decision to a different clinical context. Assessment strategies that capture both action and reasoning may therefore provide a more complete representation of competence [10,11,20].

Non-technical skills

Effective management of obstetric emergencies also depends heavily on non-technical skills. Communication, teamwork, leadership, task allocation, situational awareness, and appropriate use of available resources can influence both the efficiency and safety of emergency care. Simulation offers particular advantages for teaching these competencies because it allows students to participate in dynamic interpersonal situations that are difficult to reproduce through lectures or written clinical cases [21].

Validated instruments, such as the Team Emergency Assessment Measure (TEAM), can be used to assess non-technical performance during simulated obstetric and gynecologic emergencies [22]. Simulation-based obstetric training has also been associated with improvements in team collaboration and several non-technical domains, including communication, situational awareness, and leadership [23]. In the study by Wu et al., the median overall Clinical Teamwork Scale score increased from 5.0 before training to 7.0 after training (p = 0.002), while the median communication score increased from 5.0 to 7.0 (p = 0.001) [23]. Other studies rely on locally developed checklists, facilitator observations, or student self-evaluation, which can make comparison across interventions more difficult [10,11].

Moreover, team-based simulation introduces an additional level of complexity. A successful team outcome may not necessarily indicate that each participant has achieved an equivalent level of competence. Conversely, an individual learner may demonstrate appropriate knowledge and technical skills while team performance remains suboptimal because of communication or leadership failures [22,23]. Despite these challenges, non-technical skills should not be regarded as secondary outcomes. In real obstetric emergencies, technically correct interventions may still be delayed or ineffective if communication is poor, leadership is absent, or team members fail to establish shared situational awareness [21-23]. Consequently, comprehensive assessment of obstetric simulation should ideally incorporate both technical and non-technical dimensions of performance [21-23].

Confidence, self-efficacy, and perceived preparedness

Self-confidence, self-efficacy, perceived competence, comfort, and preparedness are among the most frequently reported outcomes following simulation-based training [5,7,8,19,20]. These variables are attractive from both educational and research perspectives because they can be measured easily using pre- and post-intervention Likert-type scales and are often responsive to simulation interventions [5,7,8,19].

Obstetric simulation studies have reported improvements across both subjective and objective educational outcomes. For example, in a cohort of 792 undergraduate medical students undergoing pig-perineum simulation training, mean self-efficacy increased from 3.63 to 4.16 on a five-point scale (p < 0.001) [8]. Simulation training has improved vaginal examination performance in medical students and team performance in obstetric emergency settings [24,25]. Increased comfort following obstetric simulation has also been reported among emergency medicine residents, while virtual-reality and task-based studies have demonstrated improvements in human, technical, and procedural skills [26-28]. The SAFE study also reported a two- to three-fold increase in self-reported confidence following high-fidelity simulation training [29]. Within undergraduate medical education specifically, improvements in confidence have been reported consistently after simulation-based training [5,7,8]. Nevertheless, confidence and competence represent conceptually distinct constructs [12].

As Gottlieb et al. emphasize, confidence is most educationally meaningful when it is appropriately calibrated with objectively demonstrated performance [12]. Both overconfidence and underconfidence may affect learning and clinical practice. Overconfidence, in particular, has been associated with diagnostic error [30].

This distinction becomes particularly important when terms such as "confidence," "perceived competence," and "clinical competence" are used interchangeably. Self-assessed competence reflects an individual's perception of their capability, whereas objective competence requires demonstrating performance against an external standard [12,20]. Therefore, studies reporting only improvements in self-confidence or perceived preparedness cannot establish that learners have acquired equivalent gains in objectively measurable clinical performance [12,20].

Recent research has increasingly attempted to evaluate both dimensions simultaneously. Studies combining subjective self-assessment with objectively measured performance suggest that simulation may improve not only competence itself but also the alignment between perceived and demonstrated ability [20]. Within obstetrics and gynecology education, emerging evidence similarly supports the importance of examining both perceived and observed performance. In a simulation curriculum involving 65 final-year medical students, median self-assessed competence increased from 50 to 54, while objectively measured skills increased from 27 to 47 (both p < 0.001). Importantly, the discrepancy between perceived and objectively assessed performance decreased to near zero after training (p < 0.001) [20]. This calibration between confidence and competence may represent an important, yet still underexplored, educational outcome in obstetric simulation.

The problem of outcome heterogeneity

Taken together, the literature uses a wide range of outcomes to define successful simulation-based learning, including knowledge scores, procedural checklists, confidence, satisfaction, teamwork, completion time, and global performance [9-11]. These measures capture different constructs and should not be treated as interchangeable. The apparent effectiveness of a simulation intervention therefore depends partly on what investigators choose to measure.

This heterogeneity becomes particularly problematic when locally developed or insufficiently validated instruments are employed. Although such tools may suit individual curricula, their widespread use reduces reproducibility and makes meaningful comparisons across studies difficult [10,11]. Composite outcome measures may introduce further complexity when knowledge, technical performance, communication, and other domains are combined into a single score without clear evidence that they represent a unified construct.

For obstetric simulation research, the more useful question is which outcomes best reflect meaningful learning and clinically relevant competence, rather than whether any post-training score improves [9-12].

A multidimensional assessment strategy appears particularly appropriate. Ideally, evaluation should integrate knowledge, objectively observed technical performance, non-technical skills, and learner self-perception, while also examining whether acquired competencies persist over time [9-12,20]. This approach provides a foundation for addressing a central question in simulation-based medical education: whether learners' confidence accurately reflects their demonstrated competence [12,20].

Confidence versus competence: are learners accurate judges of their own performance?

Confidence is commonly reported after simulation, especially in obstetrics where direct student participation may be limited [5,7,8]. It can support engagement and willingness to participate, but it remains subjective. A more informative question is whether confidence aligns with objectively demonstrated performance [31,32].

The limitations of self-assessment

The accuracy of learner self-assessment remains a persistent challenge in health professions education. A review comparing health professions students' self-assessments with objective measures of competence demonstrated that learners frequently differ from external assessors in their estimation of performance [33]. On average, students tended to overestimate their performance, although the magnitude and direction of discrepancies varied according to task characteristics and assessment methods.

In clinical education, inaccurate self-assessment can affect how learners seek supervision and take on responsibility. Overestimation may reduce recognition of personal limits, whereas underestimation may discourage appropriate participation despite adequate ability [30,33].

Recent evidence also suggests that self-assessment should not simply be viewed as an isolated judgment made by a learner. In a 2024 systematic review of self-assessment practices in medical education, Zheng et al. identified feedback from educators and peers, the format of self-assessment activities, and explicit training in self-assessment as important factors influencing how learners evaluate their own abilities [34]. Self-assessment may therefore be more educationally meaningful when embedded within a structured feedback process rather than used as an independent measure of competence.

This distinction is highly relevant when interpreting simulation studies. A post-simulation questionnaire asking students whether they feel more confident may provide valuable information regarding the learner experience, but it should not be interpreted as equivalent to external assessment of clinical ability [12,33,34]. Studies reporting exclusively subjective outcomes therefore answer a different question from studies demonstrating improved performance on a procedural checklist or structured clinical assessment [12,33].

Calibration as an educational outcome

Calibration offers a useful way to examine confidence and competence together. A well-calibrated learner is confident when performance is strong and appropriately uncertain when knowledge or skills are incomplete. Simulation is particularly suited to this analysis because self-ratings and objective performance can be collected during the same standardized encounter. Garbayo et al. paired confidence judgments with the accuracy of clinical reasoning during high-fidelity simulation, allowing learners to be classified as appropriately confident, underconfident, or overconfident [32].

This distinction matters in obstetric emergencies, where learners must make rapid decisions and also recognize when they need help. High confidence paired with an incorrect response is educationally different from the same error accompanied by appropriate uncertainty and recognition of the need for assistance [12,30,32].

Thus, confidence-competence calibration may provide information that neither confidence scores nor objective performance measures can provide on their own.

Most simulation studies are designed to demonstrate increases in knowledge, technical skills, or confidence rather than changes in calibration itself [9,12]. Consequently, an intervention may produce statistically significant increases in both confidence and performance without establishing whether the two dimensions have become better aligned [12,32].

A rise in confidence that outpaces performance improvement may indicate poorer calibration. Conversely, a temporary fall in confidence after simulation may reflect a more realistic appreciation of task complexity rather than a negative educational effect [12,32].

Recent simulation research has increasingly incorporated metacognitive approaches to explore how learners evaluate their own knowledge and performance. High-fidelity simulation has been used to compare learners' knowledge accuracy with their level of assurance and to identify gaps between what learners know and what they believe they know [32]. Such methods may be particularly valuable in high-risk specialties, where inappropriate certainty may have greater consequences than uncertainty itself.

Within obstetrics and gynecology education, emerging evidence similarly supports the importance of examining both perceived and observed performance. Recent simulation curricula involving final-year medical students have reported improvements in objective skills alongside changes in self-perceived competence, suggesting that simulation may help narrow discrepancies between learners' perceptions and externally assessed performance [20]. However, relatively few studies explicitly analyze the degree of confidence-competence alignment, leaving an important opportunity for future research [12,20,32].

The role of feedback and debriefing

Debriefing may influence not only skill acquisition but also the accuracy of subsequent self-assessment. By comparing their own interpretation of performance with facilitator observations, explicit criteria, peer feedback, or video review, learners gain external information against which self-perception can be tested [34]. Broader medical education literature likewise emphasizes structured clinical skills training, simulation, assessment, and constructive feedback in supporting learner development [35]. Within simulation settings, individualized feedback has also been associated with improved task performance [36].

For obstetric simulation, debriefing may include reviewing whether the learner correctly recognized an emergency, initiated appropriate treatment, completed critical procedural steps, communicated effectively, and escalated care at an appropriate time [14,20]. Asking learners to estimate their performance before receiving feedback may make discrepancies between self-perception and external assessment more visible [34,36].

In obstetric practice, calibration has practical consequences: learners must judge not only what should be done, but also whether they can perform it safely and when additional assistance is required [12,30,33].

An appropriately calibrated learner should ideally recognize not only what intervention is required but also whether they possess sufficient skill to perform it independently. Such awareness informs decisions about when to request senior assistance, delegate tasks, or escalate management [12,30,33].

From this perspective, overconfidence and underconfidence have different potential consequences. Overconfidence may contribute to premature autonomy and failure to seek assistance, whereas marked underconfidence may lead to hesitation or delayed intervention despite adequate competence [12,30-32]. The optimal educational outcome is therefore neither maximum confidence nor minimum uncertainty, but confidence that accurately reflects capability.

Simulation offers an opportunity to develop this calibration before students encounter equivalent situations in clinical practice. Repeated scenarios, structured assessment, deliberate practice, and feedback may enable learners to progressively refine both their clinical competence and their ability to judge that competence accurately [32,34-36].

Retention and transfer beyond the simulation setting

Most obstetric simulation studies measure outcomes immediately after training [9]. These post-test gains are useful evidence of acquisition, but they do not necessarily show that competence will persist until the learner next encounters the same procedure or emergency [9,29].

Recent evidence confirms that long-term retention remains insufficiently explored. A 2026 systematic review of simulation-based teaching for obstetric emergencies highlighted the scarcity of delayed assessments and emphasized the need for studies evaluating whether educational gains persist over time [9]. Similar concerns have been reported in broader simulation literature, where retention outcomes are assessed far less frequently than immediate learning effects [37].

Few obstetric studies have incorporated delayed reassessment. In the SAFE study, the proportion of successful simulated births increased from 8.9% before training to 93.1% after the initial workshop, while improvements in manoeuvres, communication, and confidence remained detectable at six months [29]. Greene et al. similarly evaluated procedural performance more than one month after simulation-based forceps training using an Objective Structured Assessment of Technical Skills. They found sustained improvement in both clustered and spaced training groups [38]. These findings suggest that retention can be meaningfully assessed, although evidence remains limited, particularly among undergraduate medical students.

Retention should also be distinguished according to the educational domain being measured. Persistent knowledge does not necessarily imply preservation of technical performance, while sustained confidence may not correspond to retained competence [12,29,37,38]. Ideally, delayed assessment should therefore incorporate objective performance measures alongside self-reported outcomes [29,37,38].

The question of retention is closely linked to transfer into clinical practice. Retention is only part of the problem. Even when skills persist, performance in a simulated setting does not automatically predict performance with real patients [13]. Evidence for this transfer remains limited in undergraduate obstetric education [9,13].

From a curriculum perspective, these findings favor reinforcement rather than reliance on a single simulation exposure. Although the optimal interval and training dose remain uncertain, spaced or refresher activities may help preserve competencies during the gap between simulation and clinical exposure [39]. Durable learning and transfer to practice are therefore more meaningful indicators of educational value than immediate post-training improvement alone.

Challenges and future directions in assessment

Several methodological issues still limit how confidently simulation outcomes can be interpreted. Chief among them is the wide variation in what studies measure and how those measures are constructed [9-11].

Future studies would benefit from greater use of validated tools and from pairing learner-reported outcomes with externally assessed performance. Confidence is most informative when interpreted in relation to demonstrated ability rather than as a stand-alone marker of success.

Longitudinal assessment represents another important priority. Immediate post-simulation improvement should be complemented by delayed evaluation of knowledge and skills, particularly for low-frequency obstetric emergencies. Similarly, greater emphasis should be placed on determining whether competencies acquired in simulation are transferred to authentic clinical environments.

New technologies may also support more reproducible assessment. Virtual simulation, automated performance tracking, and video-based review could provide additional information on procedural sequence, decision-making, communication, and learner progression. Their value, however, will depend on careful validation against clinically meaningful performance standards rather than on the novelty of the technology itself.

Future work should focus less on whether any outcome improves after simulation and more on whether the outcomes chosen are meaningful, durable, and transferable to clinical practice.

## Conclusions

Simulation is particularly valuable in undergraduate obstetric education because clinical exposure is variable and active participation cannot always be guaranteed. Across the literature, training is associated with gains in knowledge, procedural performance, confidence, and perceived preparedness. However, interpreting these benefits depends heavily on the outcomes used to define educational effectiveness. Confidence and self-perceived competence, although valuable, should not be considered equivalent to objectively demonstrated clinical ability. Similarly, immediate post-training performance does not necessarily establish long-term retention or successful transfer to clinical practice.

Evaluation should therefore combine learner-reported outcomes with objective performance measures, use validated tools where possible, and include delayed assessment when feasible. More meaningful evidence will come from showing that students understand their own capabilities and retain skills they can use safely beyond the simulation setting.
